# Sitagliptin therapy improves myocardial perfusion and arteriolar collateralization in chronically ischemic myocardium: A pilot study

**DOI:** 10.14814/phy2.15744

**Published:** 2023-06-10

**Authors:** Sharif A. Sabe, Dwight Douglas Harris, Mark Broadwin, Mohamed Sabra, Cynthia M. Xu, Debolina Banerjee, M. Ruhul Abid, Frank W. Sellke

**Affiliations:** ^1^ Division of Cardiothoracic Surgery, Department of Surgery, Cardiovascular Research Center, Rhode Island Hospital Alpert Medical School of Brown University, Rhode Island Hospital Providence Rhode Island USA

**Keywords:** arteriogenesis, coronary microvasculature, myocardial ischemia, sitagliptin

## Abstract

Dipeptidyl peptidase 4 inhibitors (DPP4i) may be cardioprotective based on several small animal and clinical studies, though randomized control trials have demonstrated limited benefit. Given these discrepant findings, the role of these agents in chronic myocardial disease, particularly in the absence of diabetes, is still poorly understood. The purpose of this study was to determine the effects of sitagliptin, a DPP4i, on myocardial perfusion and microvessel density in a clinically relevant large animal model of chronic myocardial ischemia. Normoglycemic Yorkshire swine underwent ameroid constrictor placement to the left circumflex artery to induce chronic myocardial ischemia. Two weeks later, pigs received either no drug (CON, *n* = 8) or 100 mg oral sitagliptin (SIT) daily (*n* = 5). Treatment continued for 5 weeks, followed by hemodynamic measurements, euthanasia, and tissue harvest of ischemic myocardium. There were no significant differences in myocardial function between CON and SIT as measured by stroke work (*p* > 0.5), cardiac output (*p* = 0.22), and end‐systolic elastance (*p* = 0.17). SIT was associated with increased absolute blood flow at rest (17% increase, IQR 12–62, *p* = 0.045) and during pacing (89% increase, IQR 83–105, *p* = 0.002). SIT was also associated with improved arteriolar density (*p* = 0.045) compared with CON, without changes in capillary density (*p* = 0.72). SIT was associated with increased expression of pro‐arteriogenic markers MCP‐1 (*p* = 0.003), TGFß (*p* = 0.03), FGFR1 (*p* = 0.002), and ICAM‐1 (*p* = 0.03), with a trend toward an increase in the ratio of phosphorylated/active PLCγ1 to total PLCγ1 (*p* = 0.11) compared with CON. In conclusion, in chronically ischemic myocardium, sitagliptin improves myocardial perfusion and arteriolar collateralization via the activation of pro‐arteriogenic signaling pathways.

## 1 INTRODUCTION

Medical therapies are limited for patients with chronic coronary artery disease with poor revascularization options (Lassaletta et al., 2011). Patients with chronic coronary disease, a leading cause of morbidity and mortality, who are poor candidates for catheter‐ and surgical‐based revascularization are at risk of developing sequelae of this disease process, including myocardial necrosis, maladaptive remodeling, and heart failure (Lassaletta et al., 2011). While several therapies are available to mitigate risk factors for coronary disease, there is an ongoing need to identify therapies that not only mitigate risk but also provide therapeutic benefit in the setting of ongoing and late‐stage coronary disease.

Recent studies have demonstrated that several antihyperglycemic agents may provide cardiovascular benefits beyond effects related to glucose control. Specifically, dipeptidyl peptidase 4 inhibitors (DPP4i) have been shown in animal studies and in some clinical studies to provide cardiovascular benefits (Bostick et al., 2014; Enzan et al., 2023; Kubota et al., 2016; Li et al., 2020; Sauvé et al., 2010; Wang et al., 2017). These agents lower glucose by inhibiting DPP4, a widely expressed proteolytic enzyme that degrades and inactivates glucose‐lowering incretins such as glucagon‐like peptide 1 and gastric inhibitory polypeptide (Scheen, 2018). In addition to these glucose‐lowering effects, DPP4i have been shown in small animal models to provide cardioprotection in the setting of acute myocardial infarction (Bostick et al., 2014; Kubota et al., 2016; Sauvé et al., 2010). Specifically, DPP4 inhibition reduced infarct size, improved cardiac function, reduced apoptosis, and attenuated decreased endothelial proliferation in a mouse model of acute myocardial ischemia (Kubota et al., 2016). Furthermore, DPP4 inhibition with sitagliptin has been shown to improve left ventricular functional recovery after myocardial ischemia/reperfusion injury in mice (Sauvé et al., 2010). In a rodent model of obesity, DPP4 inhibition improved diastolic function, reduced myocardial oxidative stress, and reduced fibrosis (Bostick et al., 2014). Therefore, DPP4i has proved cardioprotective in small animal models of acute myocardial ischemia.

Despite several cardiovascular beneficial effects of DPP4i seen in small animal studies, clinical trials have yielded mixed and largely neutral results. For instance, DPP4i have been shown to reduce progression of coronary atherosclerosis in patients with coronary disease and diabetes, (Li et al., 2020) reduce blood pressure in nondiabetic patients with hypertension,(Mistry et al., 2008) and improve long‐term outcomes in diabetes patients with heart failure and preserved ejection fraction (Enzan et al., 2023). However, large‐scale, randomized control trials of DPP4i including SAVOR‐TIMI (Udell et al., 2015), EXAMINE (White et al., 2013), TECOS (Green et al., 2015), and CARMELINA (Rosenstock et al., 2019), which investigated saxagliptin, alogliptin, sitagliptin, and linagliptin, respectively, have not demonstrated improvement in composite cardiovascular outcomes, including death from cardiovascular causes, myocardial infarction, and stroke. In fact, the SAVOR‐TIMI trial suggested an increased risk of heart failure hospitalizations with saxagliptin (Udell et al., 2015), findings that have not been reproduced in the other trials of other DPP4i agents. Of note, these trials are limited by short‐term follow‐up of typically 2–3 years (Scheen, 2018). Nonetheless, these findings highlight the lack of clarity on the effects of DPP4i in coronary disease with discrepant results between small animal studies and clinical trials.

In this study, we sought to bridge the gap between small animal and clinical trials of the effects of DPP4i on heart disease, using a large animal protocol that better models chronic myocardial ischemia than small animal trials of acute infarction, while allowing for functional and molecular investigation that can be more challenging to assess in patients. In order to assess DPP4i‐mediated effects independent of glycemic control, we utilized a nondiabetic animal model. Specifically, we aimed to determine the effects of the DPP4i sitagliptin on myocardial function, perfusion, microvessel density, and related molecular signaling in a nondiabetic swine model of chronic myocardial ischemia, in order to better understand the effects of these agents in the setting of chronic coronary disease.

## 2 METHODS

The data that support the findings of this study are available from the corresponding author upon reasonable request.

### 2.1 Ethics Statement

All experiments were approved by the IACUC of the Rhode Island Hospital (#505821), and animals were cared for in coordination with veterinary technicians at Rhode Island Hospital in compliance with the *Principles of Laboratory Animal Care* formulated by the National Society of Medical Research and the *Guide for the Care and Use of Laboratory Animals*.

### 2.2 Overview of animal model

Nineteen Yorkshire swine (Tufts) underwent thoracotomy at age 11 weeks for placement of an ameroid constrictor (Research Instruments SW) to the proximal left circumflex artery to induce chronic myocardial ischemia. Two weeks after ameroid placement, animals that survived received either 100 mg sitagliptin orally daily (“SIT,” *n* = 9, 4 male, 5 female), or vehicle (palatable food) with no drug (control, “CON,” *n* = 8, 5 male, 3 female) for a planned 5‐week course. Dosing of sitagliptin was selected based on the clinical dosing recommendation of 100 mg daily at a fixed dose. Tissue for male swine in the control group was obtained from previous experiments from an identical protocol. Five weeks after starting treatment, surviving animals in the SIT (*n* = 5, 4 male, 1 female) and CON (n = 8, 5 male, 3 female) groups underwent terminal harvest procedure for analysis. Of note, due to unexpected mortality in the SIT group (see section 3.1), the Institutional Animal Care and Use Committee (IACUC) of Rhode Island Hospital elected to hold sitagliptin treatment in two swine after 1 week, and following review and discussion of the committee, the final decision was made to continue treatment for the last week prior to terminal harvest and halt the protocol thereafter.

### 2.3 Ameroid constrictor procedure

Swine underwent ameroid constrictor placement to the proximal left circumflex artery. All pigs received oral aspirin (10 mg/kg) and oral cephalexin (30 mg/kg) preoperatively and 5 days postoperatively. A fentanyl patch (4 μg/kg) was placed preoperatively and removed 72 h later. Anesthesia was induced with telazol (4.4 mg/kg) and intramuscular xylazine (2.2 mg/kg). Pigs were intubated for mechanical ventilation, and inhaled isoflurane (0.75%–3.0% minimum alveolar concentration) was administered for anesthesia maintenance. A normal saline intravenous drip was started at a rate of 5 mL/kg/h. Swine were then positioned supine, prepped and draped in a sterile manner. A left‐sided mini‐thoracotomy was made, the pericardium was opened, and the left atrium was retracted to expose the left circumflex artery (LCx). The proximal LCx was identified near its origin from the left main coronary artery. The pig was then heparinized (80 IU/kg), and the LCx was isolated with a vessel loop. The LCx was occluded by lifting the vessel loop for 2 min, with occlusion confirmed by ST and/or T wave changes on ECG. During occlusion, 5 mL of gold microspheres (BioPal) was injected into the left atrium. The vessel loop was relaxed in order to restore blood flow to the LCx. Once ECG changes recovered to baseline, an titanium‐rim ameroid constrictor (Research Instruments SW) was sized and placed around the LCx, for gradual occlusion over 2 to 3 weeks to induce chronic myocardial ischemia (White et al., 1992). Nitroglycerin was applied topically as needed reverse vasospasm. The incision was closed in multiple layers. Amiodarone (10 mg/kg) was administered intravenously as needed for arrhythmias. Buprenorphine (0.03 mg/kg) was administered intramuscularly prior to closure.

### 2.4 Terminal harvest procedure

Five weeks after treatment, pigs underwent a terminal harvest procedure. An incision was made over the right groin for femoral artery access. Blood was collected to assess serum labs including a lipid panel and liver function tests. Then, a pressure catheter was inserted via a 6F sheath to monitor blood pressure. The heart was exposed via midline sternotomy. A butterfly needle was placed into the left atrium, and for perfusion analysis, 5 mL of isotope‐labeled microspheres (BioPal) was injected into the left atrium while 10 mL of blood was withdrawn from the femoral artery catheter simultaneously. This procedure was repeated during pacing to 150 bpm. For hemodynamic measurements and cardiac function measurements, a pressure–volume catheter (Transonic) was apically placed into the left ventricle via a 6F sheath. At the end of the procedure, anesthesia was deepened with isoflurane, and euthanasia was performed by exsanguination via excision of the heart. The myocardium was quickly divided into 16 segments based on location with respect to the left anterior descending and left circumflex arteries. Myocardial segments were air‐dried for microsphere analysis or snap‐frozen in liquid nitrogen for immunoblot analysis and frozen sectioning. The proximal circumflex artery in the area of the ameroid constrictor was inspected to confirm occlusion.

### 2.5 Myocardial perfusion analysis

Myocardial perfusion was determined using isotope‐labeled microspheres (Biophysics Assay Laboratory) injected at the time of ameroid placement and harvest. During the ameroid placement procedure, 5 mL of gold microspheres was injected into the left atrium during temporary LCx occlusion in order to determine the territory of the left ventricle perfused by the LCx. During the harvest procedure, 5 mL of Lutetium‐labeled microspheres was injected into the left atrium while simultaneously withdrawing 10 mL of blood from the femoral artery at a reference rate of 6.67 mL/min using a withdrawal pump (Harvard Apparatus). Then, the heart was paced to 150 bpm and the same injection/withdrawal protocol was repeated using samarium‐labeled microspheres. Blood samples obtained from the femoral artery withdrawal and left ventricular myocardial samples from 10 sections based on proximity of location to the left anterior descending and LCx arteries were weighed, dried, and sent to Biophysics Assay laboratory for microsphere density measurements. Blood flow was calculated using the following equation: tissue blood flow = [reference blood flow (mL/min)/tissue weight (g)] × [tissue microsphere count/reference blood microsphere count].

### 2.6 Hemodynamic/cardiac functional measurements

After obtaining femoral artery access during the harvest procedure, a pressure catheter (Transonic) was inserted into the artery and advanced into the aorta for measurement of mean arterial pressure (MAP). A pressure–volume catheter (Transonic) was then inserted into the apex of the left ventricle for cardiac functional measurements. Load‐dependent data were collected during breath holds to minimize respiratory variation, and load‐independent data were collected during breath hold and inferior vena cava occlusion. Hemodynamic data were recorded and analyzed with LabChart software (ADInstruments).

### 2.7 Immunofluorescence studies

Immunofluorescence staining was performed as described previously (Elmadhun et al., 2012). Briefly, frozen section slides were thawed, fixed with 10% paraformaldehyde, and blocked in 3% bovine serum albumin (BSA) for 1 h. Then, sections were incubated with primary antibody to α smooth muscle actin (α‐SMA; Abcam) and isolectin B4 conjugated to Alexa Fluor 647 (Thermo Fisher Scientific) overnight at 4°C. Slides were then rinsed with PBS, and anti‐mouse secondary antibody conjugated to Alexa Fluor 488 (Cell Signaling) was applied. After 1 h of incubation, slides were rinsed, and DAPI was applied for 5 min. Slides were then rinsed and mounted. Images were analyzed at 20× magnification with an Olympus VS200 Slide Scanner (Olympus Corporation). Image analysis was performed with QuPath software in a blinded fashion (Bankhead et al., 2017). Capillary and arteriolar density was determined using thresholding to define positive isolectin B4 and α‐SMA staining, respectively, and determining percent of tissue area stained.

### 2.8 Immunoblotting studies

Tissue was lysed in radioimmunoprecipitation assay buffer (Boston Bioproducts). Ischemic myocardial tissue total protein (40 μg) was fractionated on a 4%–12% Bis‐Tris gel (ThermoFisher Scientific) and transferred onto a nitrocellulose or polyvinylidene difluoride membrane (ThermoFisher Scientific). Membranes were then incubated at 4 degrees Celsius overnight with 1:1000 dilutions of individual rabbit polyclonal primary antibodies to eNOS, phosphorylated (Ser1177) eNOS (p‐eNOS), Akt, phosphorylated (Ser473) Akt (p‐Akt), extracellular signal‐regulated kinase 1/2 (ERK1/2), phosphorylated ERK1/2 (p‐ERK1/2), 5′ adenosine monophosphate‐activated protein kinase (AMPK), phosphorylated AMPK (p‐AMPK), monocyte chemoattractant protein‐1 (MCP1), transforming growth factor beta (TGFß), fibroblast growth factor receptor 1 (FGFR1), phospholipase C gamma 1 (PLCγ1), phosphorylated PLCγ1, vascular endothelial cadherin (VE‐Cadherin; Cell Signaling), endostatin, angiostatin (Abcam), intercellular adhesion molecule 1 (ICAM1), and fibroblast growth factor 1 (FGF1; Proteintech). Membranes were stripped as needed with Restore PLUS Western Blot Stripping Buffer (ThermoFisher Scientific) to allow for repeat probing. All membranes were probed with GAPDH (Cell Signaling) to correct for loading error. Membranes were then incubated with anti‐mouse or anti‐rabbit secondary antibodies (Cell Signaling), processed for chemiluminescent detection (Thermo Fisher Scientific), and captured with a digital camera system (Bio‐Rad ChemiDoc MP, Life Science). Densitometric analysis of band intensity was performed using NIH Image J software. Complete antibody catalog numbers and dilutions used are listed in Table S1.

### 2.9 Data analysis

All data are presented as median value with interquartile ranges. Immunoblot and immunofluorescence data are reported as median fold change values compared with the average control with interquartile ranges. All data were statistically analyzed with Wilcoxon rank‐sum test, using R software. Gender‐specific analysis was performed using Wilcoxon rank‐sum tests. Probability values <0.05 were considered significant.

## 3 RESULTS

### 3.1 Animal survival to harvest

Nineteen swine underwent ameroid constrictor placement. Four female pigs were allocated to the control group, of which one died intraoperatively during ameroid placement following severe bradycardia, ventricular fibrillation, and cardiac arrest (20% mortality). Five male pigs were included in the control group based on tissue availability from a cohort that had a 17% mortality rate. Of the 10 pigs allocated to the sitagliptin arm of the study, there were a total of five deaths (four females, one male, 50% mortality). One male pig was found dead on postoperative day (POD) 1 with necropsy‐based cause of death suspected as tamponade. Three female pigs were found dead in their cage several weeks after ameroid placement (POD 24, POD 27, POD 39), with unclear etiology on necropsy and presumed sudden cardiac arrest. One female pig was found on POD17 to be lethargic, tachypneic, mouth breathing, with normal vital signs, and due to veterinary team concern, the pig was euthanized. Necropsy and lab work in this pig were unremarkable for etiology of symptoms. There was therefore a trend toward increased mortality in the sitagliptin arm of the study when considering the mortality rate of historic controls (Fisher's exact test *p* = 0.10).

### 3.2 Metabolic parameters

There were no differences in body mass index (BMI) or BMI percent change from ameroid to harvest procedures between the control and sitagliptin‐treated swine. Sitagliptin‐treated swine had increased serum levels of alkaline phosphatase, decreased alanine aminotransferase, decreased triglycerides, and increased low‐density lipoprotein compared with control. There were no significant differences between groups in serum levels of albumin, total protein, aspartate aminotransferase, total cholesterol, high‐density lipoprotein, C reactive protein, and hemoglobin A1c (Table 1).

**TABLE 1:** Metabolic parameters.

Metabolic parameter	Control	IQR	Sitagliptin	IQR	*p* value
BMI at ameroid (kg/m^2^)	58.8	52.2, 66.2	61.5	44.9, 64.3	0.62
BMI at harvest (kg/m^2^)	64.4	56.7, 71.4	76.7	73.7, 77.4	0.09
% Change BMI	6.4	−1.9, 13.6	25.7	11.6, 42.0	0.28
Albumin (g/dL)	3	2, 3	3	2, 3	0.61
Total protein (g/dL)	5	5, 5	5	5, 5	0.50
AST (IU/L)	19	18, 24	16	15, 19	0.24
ALT (IU/L)	53	52, 56	20	19, 28	0.004
Alkaline phosphatase (IU/L)	135	119, 152	201	176, 205	0.019
Total bilirubin (mg/dL)	0.1	0.1, 0.1	0.1	0.1, 0.1	1
Total cholesterol (mg/dL)	70	58, 77	75	73, 75	0.61
HDL (mg/dL)	37	32, 42	34	34, 35	0.42
LDL (mg/dL)	30	21, 34	41	39, 41	0.012
Triglycerides (mg/dL)	17	15, 22	10	10, 14	0.025
CRP (mg/L)	0.20	0.20, 0.36	0.20	0.20, 0.34	0.93
Glucose (mg/dL)	172	157, 209	166	150, 171	0.34
HbA1c (%)	<4	—	<4	—	1

*Note*: Statistics presented as median values with interquartile ranges (IQR). *p* values calculated with Wilcoxon rank‐sum test.

Abbreviations: ALT, alanine aminotransferase; AST, aspartate aminotransferase; BMI, body mass index; CRP, C reactive protein; HbA1c, hemoglobin A1c.; HDL, high‐density lipoprotein; LDL, low‐density lipoprotein.

#### 3.2.1 Sitagliptin does not improve cardiac function in the setting of chronic myocardial ischemia

**FIGURE 1:**
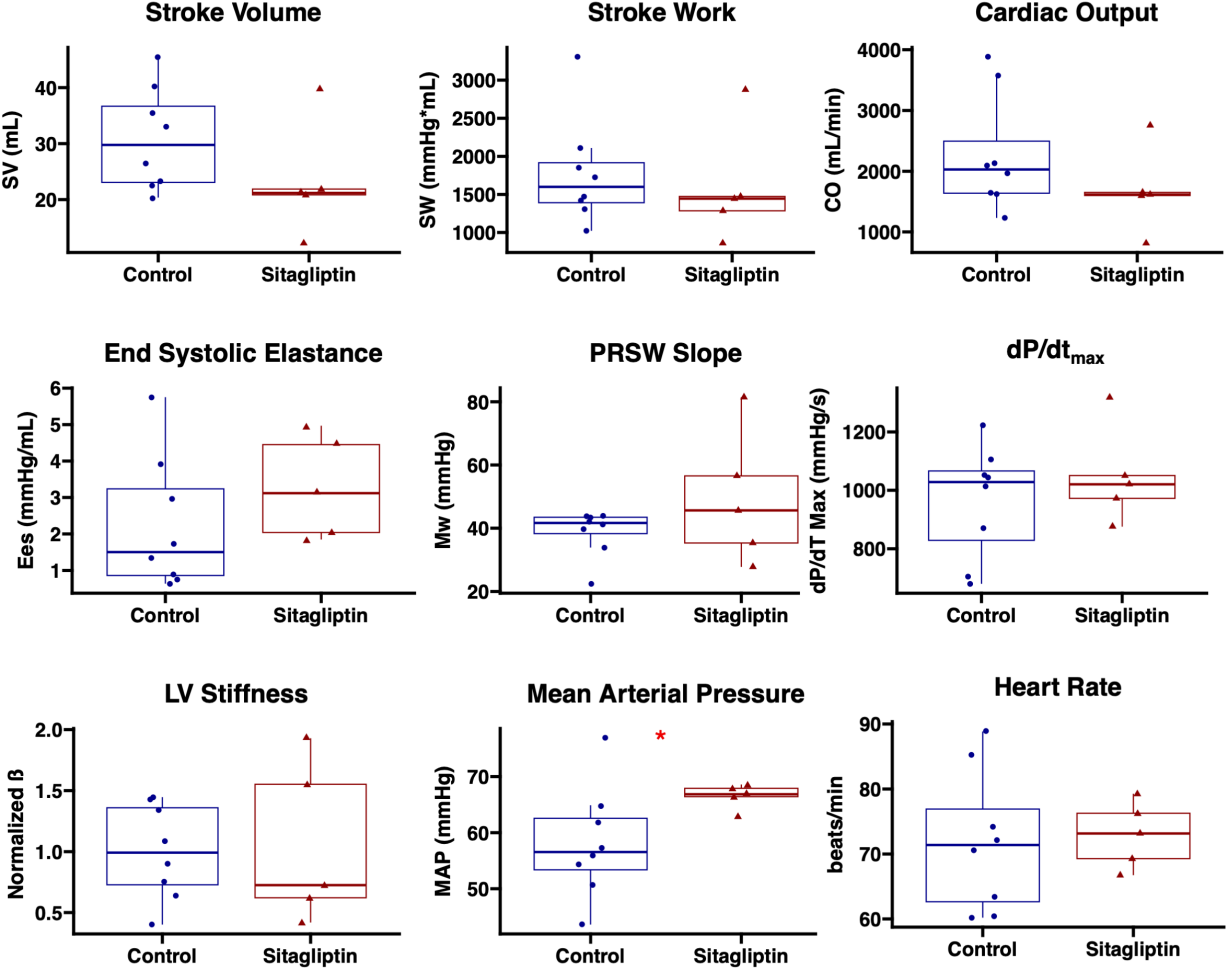
Sitagliptin does not improve cardiac function in the setting of chronic myocardial ischemia. Swine treated with sitagliptin (*n* = 5, 4 male, 1 female) had trends toward reduced stroke volume, stroke work, and cardiac output compared with controls (*n* = 8, 5 male, 3 female). There were no differences in contractility as measured by end‐systolic elastance, the slope of the preload recruitable stroke work (PRSW) relationship, or d*P*/d*t*_max_. There were no differences in left ventricular (LV) stiffness as determined by normalized ß as derived from the end‐diastolic pressure–volume relationship. The sitagliptin‐treated group had increased mean arterial pressure without differences in heart rate. *p < 0.05. Data analyzed using Wilcoxon rank‐sum test.

Swine in the sitagliptin‐treated group had higher mean arterial blood pressure at the time of the harvest procedure compared with control (*p* = 0.045), without differences in heart rate (*p* = 0.72). There were no significant differences in myocardial function between CON and SIT groups as measured by stroke work (*p* = 0.52), stroke volume (*p* = 0.13), and cardiac output (*p* = 0.22), though there was a decreased trend in the latter two parameters in the SIT group. There were no differences in contractility between groups as measured by d*P*/d*t*_max_ (*p* = 0.72), end‐systolic elastance (*p* = 0.17), and the slope of the preload recruitable stroke work relationship (*p* = 0.35). There were no differences in normalized left ventricular stiffness coefficient, as derived from the end‐diastolic pressure–volume relationship, between groups (*p* = 1). There were no significant differences in cardiac function parameters when analysis was separated by gender (Figure 1, Table 2, Figure S1).

**TABLE 2:** Hemodynamic parameters.

Parameter	Control	IQR	Sitagliptin	IQR	*p* value
HR (beats/min)	71.4	62.7, 76.9	73.2	69.3, 76.3	0.72
Systolic BP (mm Hg)	68.6	65.5, 84.4	83.6	81.0, 86.4	0.17
Diastolic BP (mm Hg)	44.7	43.6, 47.2	53.0	51.4, 55.2	0.034
MAP (mm Hg)	56.6	53.4, 62.6	66.9	66.4, 67.9	**0.045**
SV (mL/beat)	29.8	23.1, 36.7	21.2	20.8, 21.9	0.13
SW (mm Hg × mL)	1599.3	1391.8, 1916.5	1445.3	1286.0, 1473.7	0.52
CO (mL/min)	2029.5	1638.3, 2495.5	1615.7	1600.3, 1652.0	0.22
d*P*/d*t*max	1028.1	828.8, 1066.4	1020.3	972.4, 1050.3	0.72
d*P*/d*t*min	−1402.8	−1625.1, −1116.0	−1407.7	−1410.5, −1395.5	0.94
Ees (mm Hg/mL)	1.5	0.9, 3.2	3.1	2.0, 4.5	0.17
Slope_PRSW_ (mm Hg)	41.6	38.3, 43.4	45.6	35.3, 56.5	0.35
Normalized ß	1.0	0.7, 1.4	0.7	0.6, 1.6	1.00

*Note*: Statistics presented as median values with interquartile ranges (IQR). *p* values calculated with Wilcoxon rank‐sum test. Bold indicates significant values.

Abbreviations: BP, blood pressure; CO, cardiac output; d*P*/d*t*, change in pressure over change in time; Ees, end‐systolic elastance; HR, heart rate; MAP, mean arterial pressure; PRSW, preload recruitable stroke work relationship; ß, left ventricular stiffness coefficient as derived by the end‐diastolic pressure–volume relationship; SV, stroke volume; SW, stroke work.

#### 3.2.2 Sitagliptin improves myocardial perfusion to ischemic territory

**FIGURE 2:**
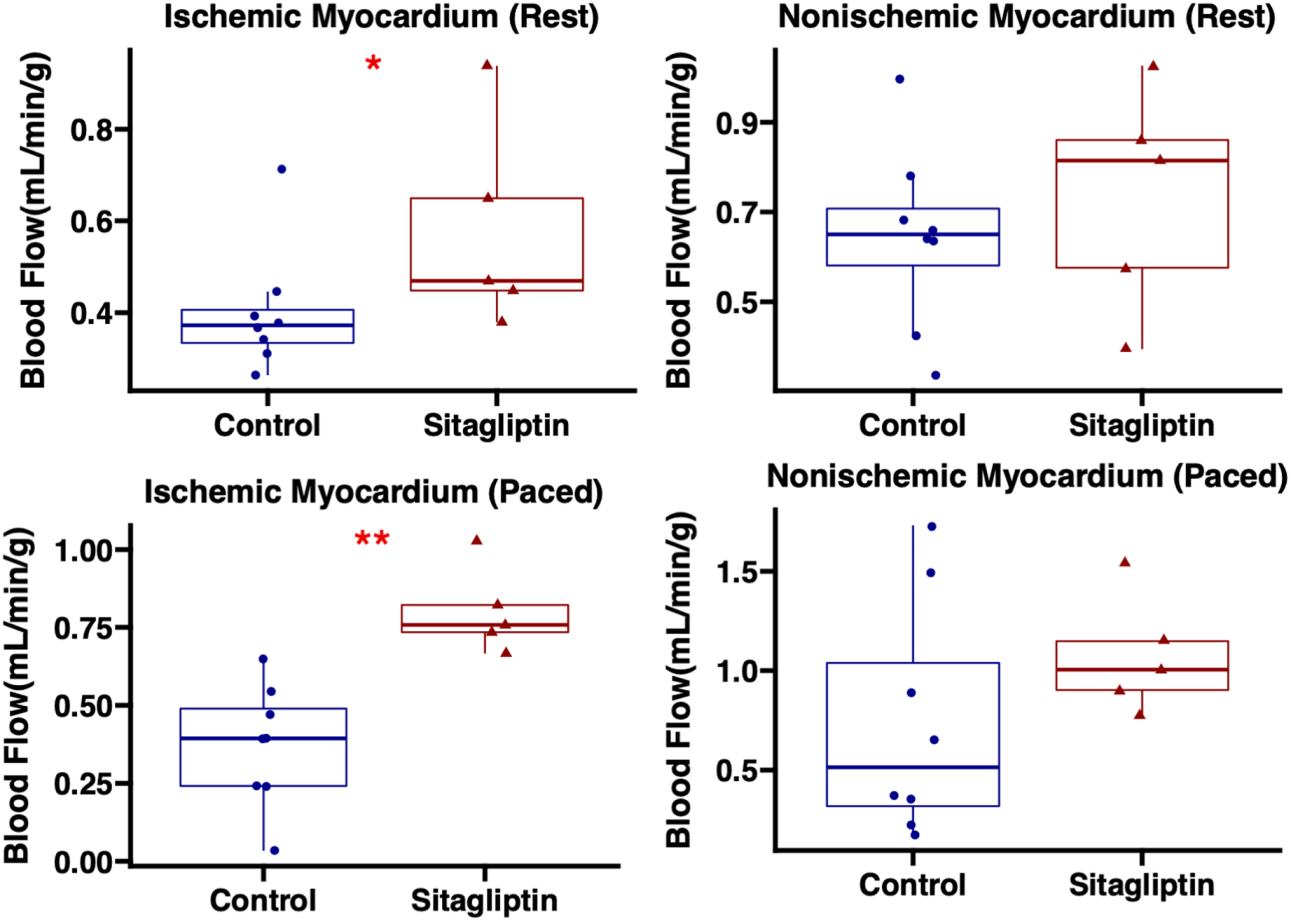
Sitagliptin improves myocardial perfusion to ischemic territory. Swine treated with sitagliptin (*n* = 5, 4 male, 1 female) had increased absolute blood flow to chronically ischemic myocardium compared with control (*n* = 8, 5 male, 3 female) as measured by microsphere analysis. There were no significant differences in perfusion to nonischemic territory between groups. *p < 0.05, **p < 0.01. Data analyzed using Wilcoxon rank‐sum test.

Sitagliptin therapy was associated with improved myocardial perfusion to ischemic myocardial territory both at rest (17% increase, *p* = 0.045) and during pacing (89% increase, *p* = 0.002). There were no significant differences in perfusion to nonischemic myocardial territory at rest (*p* = 0.52) or during pacing (*p* = 0.17; Figure 2). Among male swine, there was increased perfusion to ischemic territory with sitagliptin during pacing (*p* = 0.016), and a trend toward increased perfusion to ischemic territory at rest (*p* = 0.063). There was also an increase in perfusion in sitagliptin‐treated males to nonischemic territory during pacing (*p* = 0.016; Figure S1).

#### 3.2.3 Sitagliptin increases arteriolar density to ischemic myocardial territory

**FIGURE 3:**
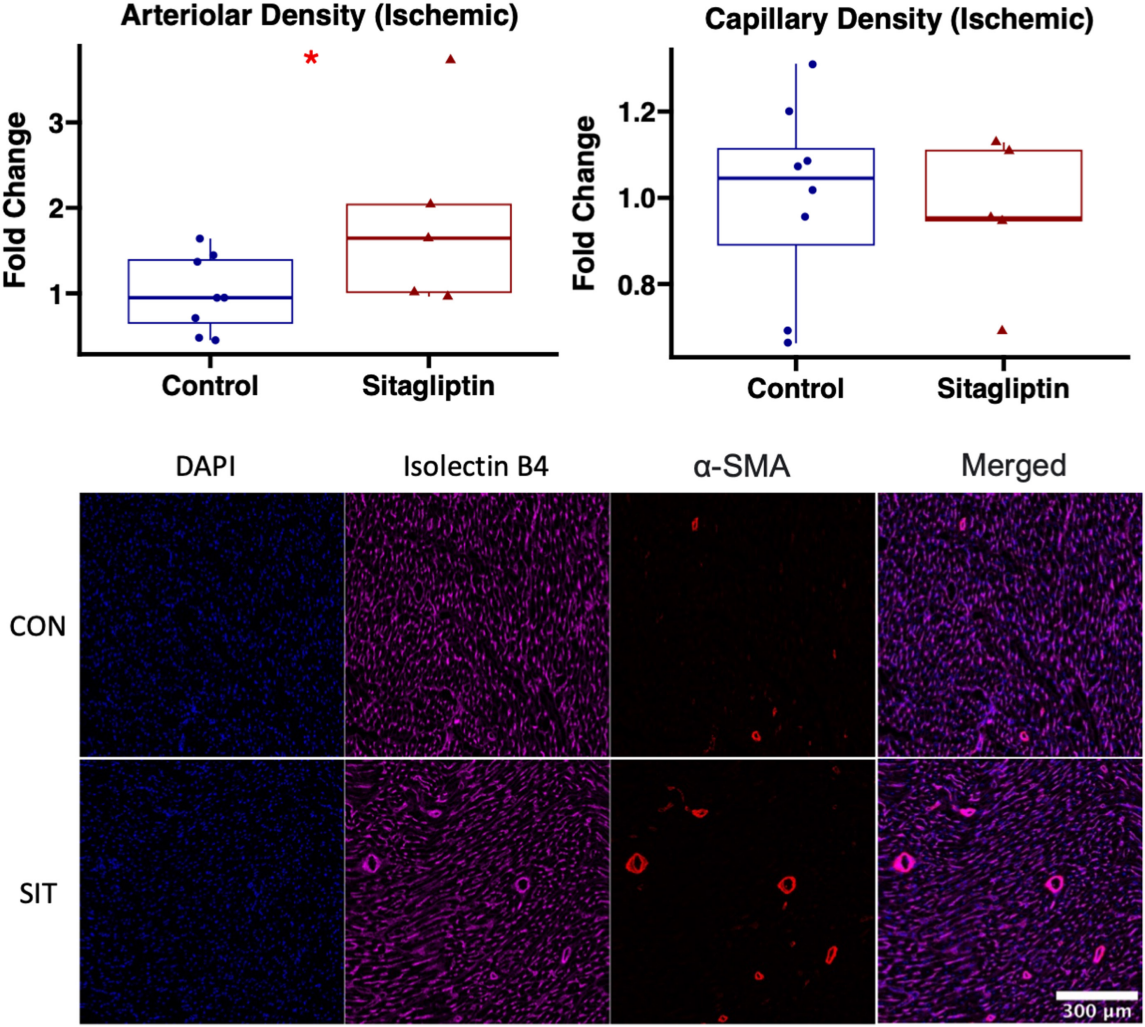
Sitagliptin increases arteriolar density to ischemic myocardial territory. Sitagliptin‐treated swine (SIT, *n* = 5, 4 male, 1 female) had increased arteriolar density in chronically ischemic myocardium compared with control (CON, *n* = 8, 5 male, 3 female). There were no differences in capillary density between groups. Representative images shown of ischemic myocardial tissue stained with alpha‐smooth muscle actin (α‐SMA) for arteriolar density (red) and isolectin B4 for capillary density (purple). *p < 0.05. Data analyzed using Wilcoxon rank‐sum test.

In ischemic myocardial territory, sitagliptin therapy was associated with improved arteriolar density as measured by α‐SMA immunostaining, compared with control (*p* = 0.045). There were no differences in capillary density, as measured by isolectin B4 immunostaining, between groups (*p* = 0.72; Figure 3). Among males, there was a trend toward increased arteriolar density in sitagliptin‐treated swine compared with control (*p* = 0.063), though this did not reach statistical significance due to low sample size (Figure S1).

#### 3.2.4 Sitagliptin upregulates pro‐arteriogenic signaling markers

**FIGURE 4:**
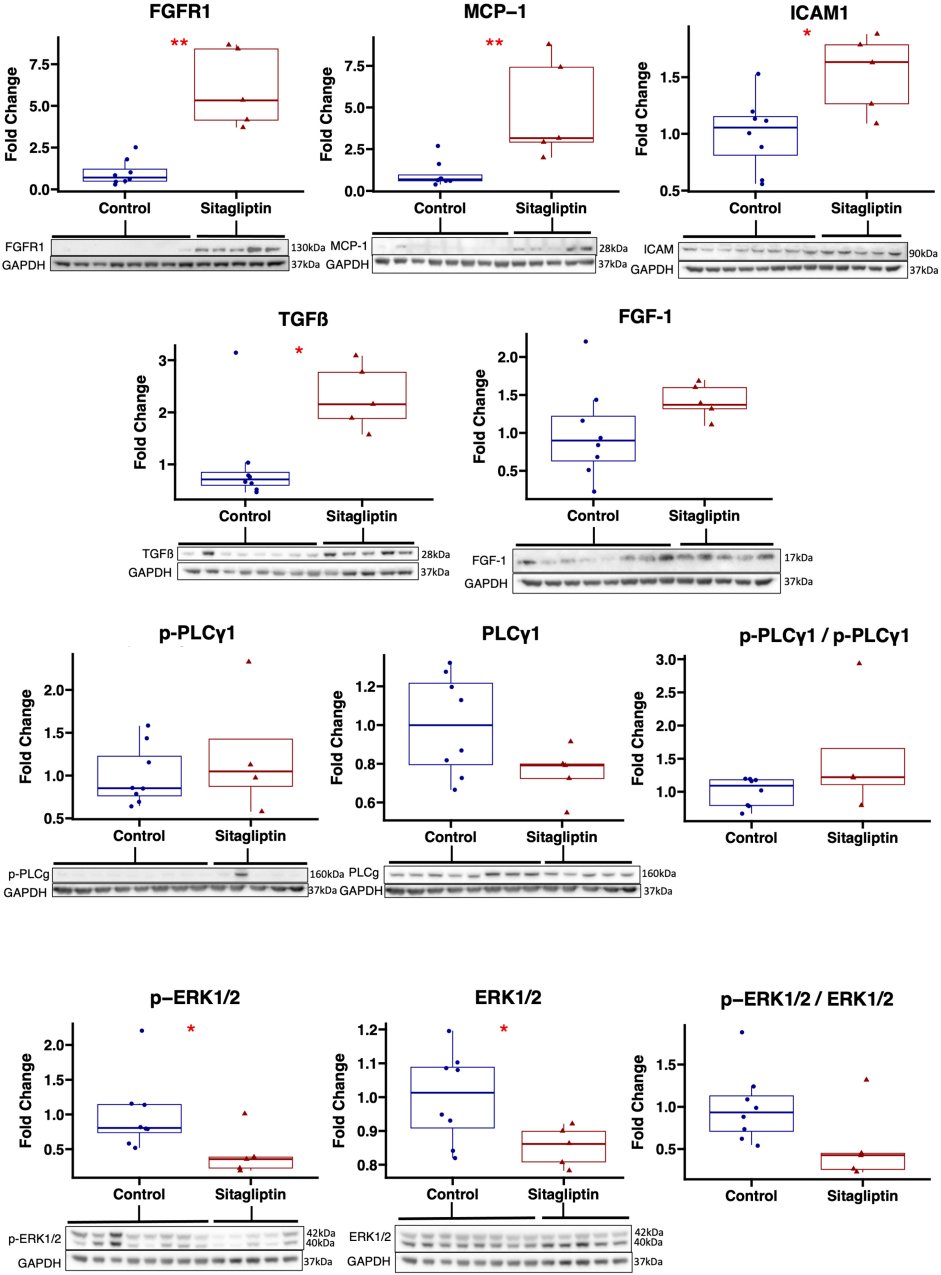
Sitagliptin upregulates several pro‐arteriogenic signaling markers in chronically ischemic myocardium. Swine treated with sitagliptin (*n* = 5, 4 male, 1 female) had upregulation of several pro‐arteriogenic markers in chronically ischemic myocardial territory compared with control (*n* = 8, 5 male, 3 female), including fibroblast growth factor receptor 1 (FGFR1), monocyte chemoattractant protein‐1 (MCP‐1), intercellular adhesion molecule 1 (ICAM1), transforming growth factor beta (TGFß), with a trend toward increased fibroblast growth factor 1 (FGF‐1) and the ratio of phosphorylated (p‐) phospholipase C gamma 1 (PLCγ1) to total PLCγ1. Phosphorylated and total levels of extracellular signal‐regulated kinase ½ (ERK1/2) were decreased in the sitagliptin group compared with control. Blots for p‐PLCγ1 and p‐ERK1/2 were stripped and re‐probed with PLCγ1 and ERK1/2, respectively, in order to calculate ratios. The same respective blots were re‐probed for GAPDH as a loading control; therefore, the same GAPDH blot panel is shown under each test blot panel. Results for FGF‐1 and ICAM1 were obtained by probing the same blot; therefore, the same GAPDH blot panel is shown under each respective test blot panel. *p < 0.05, **p < 0.01. Data analyzed using Wilcoxon rank‐sum test.

In ischemic myocardium of the sitagliptin‐treated group, there was increased expression of pro‐arteriogenic markers monocyte chemoattractant protein‐1 (*p* = 0.003), transforming growth factor beta (*p* = 0.03), fibroblast growth factor receptor 1 (*p* = 0.002), and intercellular adhesion molecule 1 (*p* = 0.03), with a trend toward an increase in the ratio of phosphorylated/active phospholipase C gamma 1 to total phospholipase C gamma 1 (*p* = 0.11, one outlier excluded), and increased fibroblast growth factor 1 (*p* = 0.13) compared with control. There was decreased expression of p‐ERK1/2, total ERK1/2 (*p* = 0.045), and a trend toward decreased ratio of phosphorylated to total ERK1/2 (*p* = 0.065; Figure 4).

**FIGURE 5:**
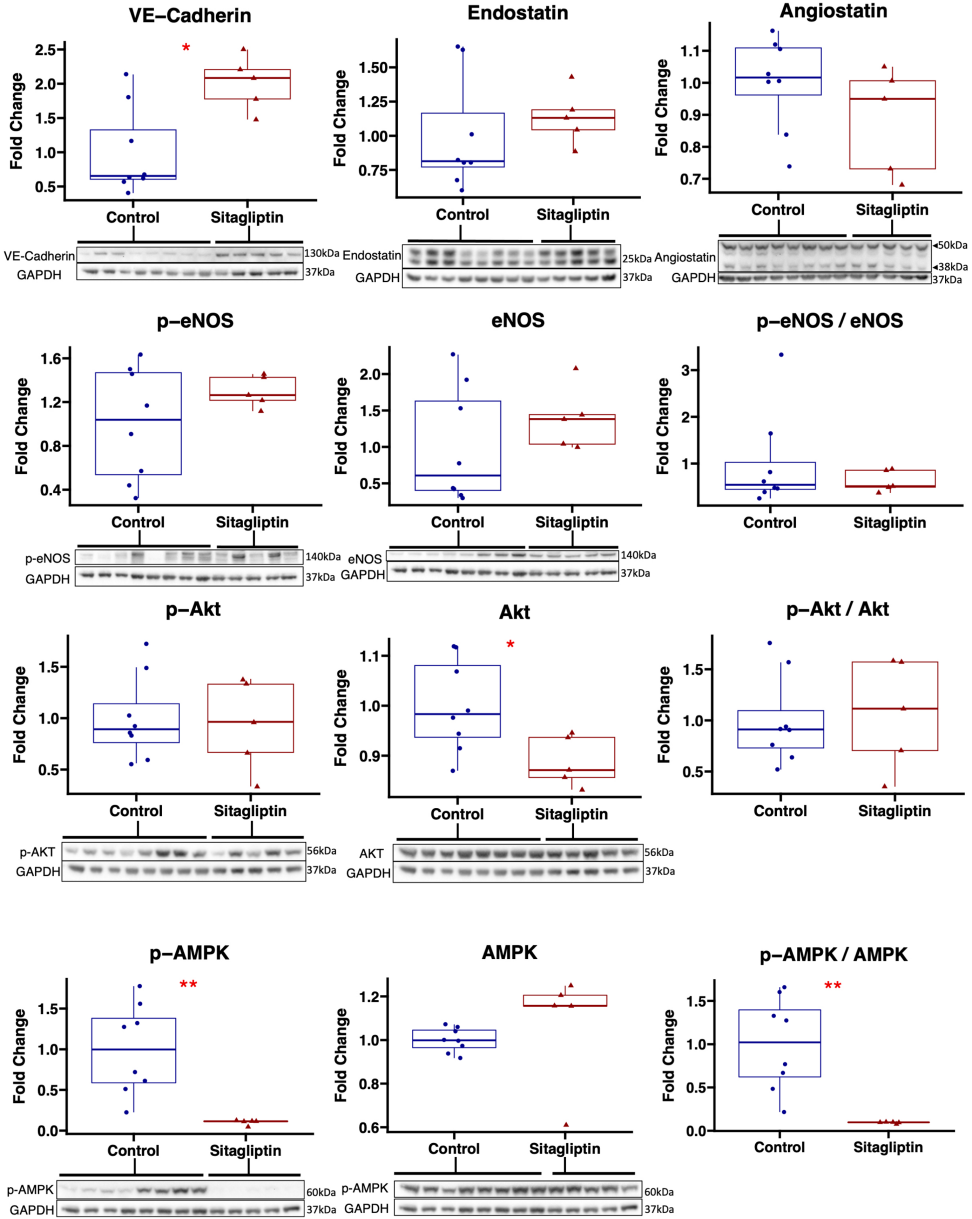
Effects of sitagliptin on angiogenic signaling markers in chronically ischemic myocardium. Swine treated with sitagliptin (*n* = 5, 4 male, 1 female) had increased expression of vascular endothelial cadherin (VE‐Cadherin) in ischemic myocardium compared with control (*n* = 8, 5 male, 3 female), without differences in expression endostatin, angiostatin, phosphorylated (p‐) endothelial nitric oxide synthase (eNOS), total eNOS, or the ratio of p‐eNOS to eNOS. Total Akt expression was decreased in the sitagliptin‐treated group without differences in p‐Akt or the ratio of p‐Akt to total Akt. Phosphorylated 5′ adenosine monophosphate‐activated protein kinase (AMPK) and the ratio of p‐ to total AMPK was decreased in the sitagliptin‐treated group compared with control. Blots for p‐AMPK, p‐eNOS, and p‐Akt, 1 were stripped and re‐probed with AMPK, eNOS, and Akt, respectively, in order to calculate ratios. The same respective blots were re‐probed for GAPDH as a loading control; therefore, the same GAPDH blot panel is shown under each test blot panel.*p < 0.05, **p < 0.01. Data analyzed using Wilcoxon rank‐sum test.

Immunoblotting for other important angiogenic markers showed that sitagliptin‐treated swine had increased expression of vascular endothelial cadherin (*p* = 0.03) in ischemic myocardial territory. There was decreased expression of total Akt (*p* = 0.045) in the sitagliptin‐treated group but without differences in expression of activated Akt (*p* = 0.94) or the ratio of phosphorylated to total Akt (0.83). There were no differences in expression of total eNOS (*p* = 0.35), phosphorylated eNOS (*p* = 0.62), or the ratio of phosphorylated to total eNOS (*p* = 0.94) between groups. There were no differences in expression of endostatin (*p* = 0.22), or angiostatin (*p* = 0.22) between groups. There was decreased expression of phosphorylated/active AMPK and decreased ratio of phosphorylated to total AMPK in the sitagliptin‐treated group compared with control (Figure 5). Complete blot images are shown in Figure S2.

## 4 DISCUSSION

In the present study, we found that in a swine model of chronic myocardial ischemia, sitagliptin therapy is associated with improved perfusion to ischemic territory, which may be secondary to increased arteriolar collateralization with upregulation of pro‐arteriogenic signaling pathways. Interestingly, changes were not associated with improvement in cardiac function. These findings may provide important insights about cardiac‐specific effects of DPP4i.

Several recent antihyperglycemic agents have recently garnered attention for providing cardiovascular benefits beyond effects related to glucose control, including sodium–glucose transporter 2 inhibitors, glucagon‐like peptide 1 receptor agonists, and dipeptidyl peptidase 4 inhibitors. Despite several randomized clinical trials demonstrating clinical benefit for the former two agents in patients with cardiovascular disease (Das et al., 2020), the lack of cardiovascular benefit of DPP4i in clinical trials has resulted in mixed opinions regarding the role of these agents in patients with diabetes and cardiovascular disease (Scheen, 2018). Nonetheless, many patients with diabetes and concurrent coronary disease are taking DPP4i, underscoring the clinical importance of investigating the effects of these agents in the setting of myocardial disease.

In the setting of coronary disease and chronic myocardial ischemia, collateralization via capillary proliferation and/or arteriolar development may mitigate ischemic injury (Lavine et al., 2013; Rezende et al., 2019). In our model of chronic myocardial ischemia, we found that sitagliptin treatment was associated with increased arteriolar density in chronically ischemic territory. In mouse models, sitagliptin improved capillary collateralization in the setting of hind limb ischemia (Dai et al., 2018), and DPP4 inhibition has been shown to attenuate endothelial cell loss in the heart following acute myocardial infarction in mice (Kubota et al., 2016). In contrast, we did not see any evidence of increased capillary collateralization in ischemic territory, which may be secondary to differences in disease course (acute infarction vs chronic ischemia), differences in treatment duration (days vs weeks), and differences in responses between species (rodents vs swine). Furthermore, increased arteriogenesis with DPP4 inhibition has been seen in hind limb ischemia animal models (Vedantham et al., 2018), though similar studies in coronary circulation in the setting of myocardial ischemia are lacking. This study is therefore the first to our knowledge to investigate and demonstrate increased coronary arteriogenesis in the setting of myocardial ischemia. The improved arteriogenesis may have contributed to our finding of improved perfusion to the ischemic myocardium. Although studies on coronary perfusion with DPP4i are lacking, there are some clinical studies that suggest DPP4 inhibition may improve coronary flow reserve and myocardial perfusion reserve in patients with diabetes and CAD (Kato et al., 2016; Oh et al., 2021). Our microsphere analysis is a more direct measure of perfusion, so this study may provide further evidence that sitagliptin improves myocardial blood flow.

With occlusion of a coronary vessel, increased flow to collateral vessels stimulates MCP‐1 and ICAM expression from endothelial cells (Carmeliet, 2000). Subsequent macrophage recruitment to the media of the vessel wall and release of growth factors results in the degradation and re‐synthesis of the smooth muscle layer of the vessel wall which ultimately results in arteriogenesis (Carmeliet, 2000). We found that in chronically ischemic myocardium, there was increased expression of many of these necessary markers, including macrophage recruiting markers MCP‐1 and ICAM, as well as growth factor‐related markers including FGFR1 and TGFß, with a trend toward increased FGF‐1 expression. There was also a trend toward increased activation of PLCγ1 with sitagliptin therapy, an important mediator of arteriogenesis (Ren et al., 2010). Interestingly, ERK1/2 expression and activation was decreased with sitagliptin, though this did not impair arteriolar proliferation. Therefore, sitagliptin upregulated pro‐arteriogenic markers that are necessary in the myocardium's adaptive response to chronic ischemic injury.

Several previous small animal studies have indicated that DPP4i have cardioprotective effects, particularly with regard to cardiac function, in the setting of acute myocardial infarction and ischemia/reperfusion. In a mouse model of acute myocardial infarction, Kubota and others found that DPP4 inhibition reduced infarct size, improved cardiac function, and reduced apoptosis (Kubota et al., 2016). Sauvé et al. (2010) found that sitagliptin reduced mortality in diabetic mice with myocardial infarction and improved left ventricular functional recovery after ischemia/reperfusion injury. Additionally, Bostick and others found that DPP4 inhibition in obese mice improved diastolic function and reduced myocardial oxidative stress and fibrosis (Bostick et al., 2014). Importantly, all of these small animal studies showed improved cardiac functional recovery with DPP4 inhibition in the setting of acute myocardial ischemia, yet this effect was not seen in the current study within our swine model of chronic myocardial ischemia. Rather, there were trends toward reduced stroke volume, stroke work, and cardiac output in the sitagliptin‐treated group, with a trend toward increased overall mortality. These findings may be more consistent with the negative results from some clinical trials on cardiovascular outcomes with DPP4 inhibitors, including some studies suggesting worsening cardiac function and heart failure hospitalizations with DPP4i (Packer, 2018a; Udell et al., 2015). Therefore, the cardiac functional effects of sitagliptin demonstrated in this study may be more consistent with results from clinical trials, possibly due to the increased clinical relevance of our swine model compared with small animal models. Trends toward reduced cardiac function and increased mortality despite improved perfusion and collateralization to the ischemic area suggest that there may be other deleterious molecular effects that are offsetting many of the benefits that we saw in this study. One such molecular effect may be decreased activation of AMPK, which is a central mediator of several important processes including cardiac metabolism, gene transcription, mitochondrial function, and contractility (Li et al., 2019; Young & Zaha, 2012). Decreased AMPK may mediate reduced cardiac function, and it is possible severe reduction in cardiac function led to the late deaths in the sitagliptin‐treated arm. There are other studies to suggest that DPP4i may potentiate mediators of increased sympathetic activation and ß‐adrenergic receptor stimulation which may worsen cardiac function (Packer, 2018b), and further investigation in this area using our model may be useful, particularly in the setting of simultaneous ß‐blockade and DPP4 inhibition.

Interestingly, the swine treated with sitagliptin had increased MAP compared with control. Studies have shown that DPP4i typically either have no effect on blood pressure or slightly decrease blood pressure (Zhang et al., 2019), though importantly these studies have typically been performed in patients with diabetes and hypertension, while the swine in our model were nondiabetic and normotensive. The known effects of sitagliptin on increasing circulating catecholamines could have a more pronounced effect on blood pressure in otherwise normotensive swine (Wilson et al., 2022), though further studies in this area may clarify these findings.

Sex‐specific effects of sitagliptin would be difficult to statistically assess in our study given low sample size; however, the high mortality among particularly the female treated swine was notable. Of the five deaths in the sitagliptin arm of the study, four of those deaths were late deaths in the female swine, while the male death was on POD1 and likely due to an acute postoperative complication. Female sex is generally considered cardioprotective in the setting of myocardial ischemia (Ostadal & Ostadal, 2014), yet the one female treated pig that survived had decreased stroke volume, stroke work, and cardiac output compared with female controls. Sex‐specific analyses on the cardiovascular effects of DPP4 inhibition are limited, and although there are no clinical data suggesting major differences in outcomes between men and women on DPP4i (Raparelli et al., 2020), there are data to suggest sex‐based differences in DPP4 activity in relation to cardiovascular risk (Pérez‐Durillo et al., 2018). Further studies are necessary to investigate sex‐specific effects of DPP4 inhibition in myocardial ischemia, and whether sex‐hormone interactions may play a role.

There are several important limitations in this study to consider. One limitation is low sample size, particularly in the sitagliptin‐treated group (*n* = 5), and some areas of the study may have been underpowered for detection of differences, particularly given the trends toward reduced cardiac function and increased mortality. This limitation was particularly notable for our gender‐specific analysis, as there was only one female pig in the sitagliptin‐treated group that survived. Resource limitations in our large animal protocol also required that we include control tissue from previous experiments, though these experiments were performed at the same facility, with the same animal team and protocol in order to minimize variability. A major limitation was also the incomplete treatment course of sitagliptin in two of the swine due to unexpected mortality and halting of the protocol by the IACUC. Despite these pigs not receiving a full course of treatment, 2 weeks of sitagliptin therapy appeared to still have similar effects as 5 weeks of therapy in the other treated swine with regard to cardiac perfusion and arteriolar density, though indices of cardiac function were slightly higher in these two animals compared with the three that received full treatment. These findings may have implications on the necessary duration of sitagliptin therapy for effective changes in ischemic myocardium. Another limitation is the lack of sufficient cardiovascular monitoring in swine following initiation of therapy in order to better understand the cause of death in the sitagliptin‐treated swine. Swine are monitored for basic activity and vital signs at regular intervals, but not continuously, and most of the deaths occurred in between monitoring intervals. Therefore, cause of death was not certain and often presumed to be dysrhythmia, though continuous electrocardiogram monitoring devices could be useful in future studies for better clarity. Finally, though the focus of our molecular investigation was on angiogenic and arteriogenic signaling pathways, there are likely other myocardial molecular pathways involved with sitagliptin therapy that may offset some of the beneficial changes we saw with regard to perfusion and arteriogenesis. Therefore, further studies will be warranted to better understand the effects of sitagliptin in the setting of chronic myocardial ischemia. As previously discussed, we utilized a model of nondiabetic swine in order to determine the effects of DPP4i independent of glucose control, though in the future we plan to utilize a large animal model of metabolic syndrome in order to better understand the effects of these agents in a more clinically relevant context.

## 5 CONCLUSIONS

In the absence of diabetes, sitagliptin therapy improves myocardial perfusion to chronically ischemic myocardial territory, with increased arteriolar collateralization and activation of pro‐arteriogenic signaling pathways. Although these benefits did not translate to improvements in myocardial function, further investigation into the mechanistic effects of DPP4 inhibitors in the setting of chronic coronary disease may help develop therapeutic modalities.

## FUNDING INFORMATION

This research was funded by the National Heart, Lung, and Blood Institute (NHLBI) 1F32HL160063–01 (S.A.S.); T32 GM065085 [J.A.](C.M.X., D.B.); NIH T32HL160517 [F.W.S] (D.D.H., M.B.); 1R01HL133624 (M.R.A.); 2R56HL133624–05 (M.R.A.); R01HL46716 and R01HL128831 (F.W.S.).

## CONFLICT OF INTEREST STATEMENT

None.

## Supporting information


Figure S1.

Figure S2.

Table S1.
