# Supplementary figures and images for: Sitagliptin therapy improves myocardial perfusion and arteriolar collateralization in chronically ischemic myocardium: A pilot study
Source: Physiol Rep. 2023 Jun 10;11(11):e15744. doi: 10.14814/phy2.15744 (PMC10257079; doi:10.14814/phy2.15744)

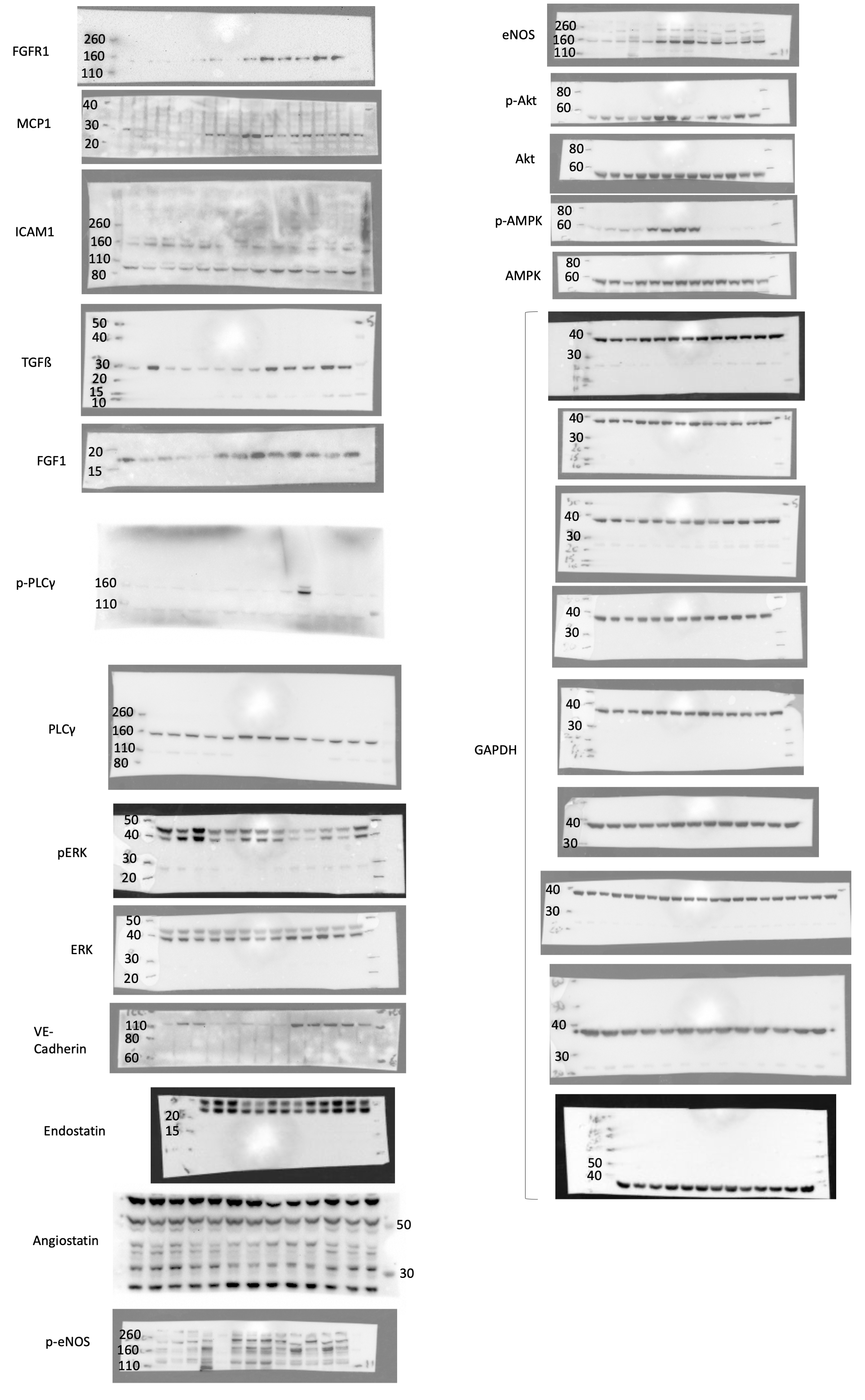

Supplement: Supplementary file 1 — Figure S1. [file PHY2-11-e15744-s003.jpg]

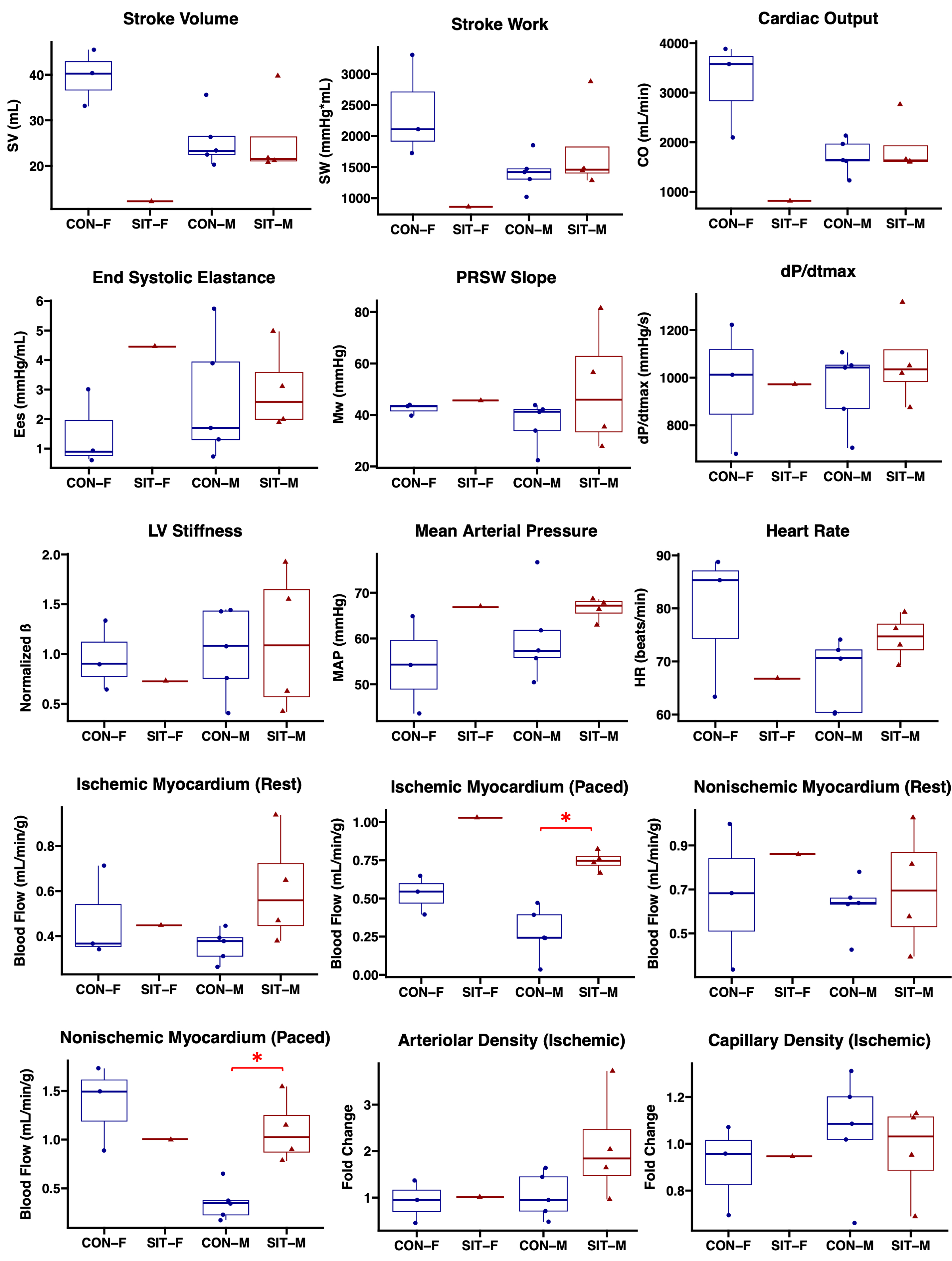

Supplement: Supplementary file 2 — Figure S2. [file PHY2-11-e15744-s002.jpg]
