# Supplementary material for: Sitagliptin therapy improves myocardial perfusion and arteriolar collateralization in chronically ischemic myocardium: A pilot study
Source: Physiol Rep. 2023 Jun 10;11(11):e15744. doi: 10.14814/phy2.15744 (PMC10257079; doi:10.14814/phy2.15744)
Supplement: Supplementary file 3 — Table S1. [file PHY2-11-e15744-s001.docx]

| **Antibody Name** | **Manufacturer** | **Catalog Number** | **Concentration** |
| --- | --- | --- | --- |
| Akt | Cell Signaling | 9272 | 1:1000 |
| AMPK | Cell Signaling | 2532 | 1:1000 |
| Angiostatin | Abcam | 2904 | 1:500 |
| Anti-rabbit IgG, HRP-linked Antibody | Cell Signaling | 7074 | 1:4000 |
| Anti-mouse IgG, HRP-linked Antibody | Cell Signaling | 7076 | 1:4000 |
| Anti-mouse IgG (H+L) Fragment – Alexa Fluor 488 Conjugate | Cell Signaling | 4408 | 1:200 |
| Endostatin | Abcam | 207162 | 1:1000 |
| eNOS | Cell Signaling | 32027 | 1:1000 |
| ERK1/2 | Cell Signaling | 4695 | 1:2000 |
| FGF1 | Proteintech | 17400 | 1:1000 |
| FGFR1 | Cell Signaling | 9740 | 1:1000 |
| GAPDH | Cell Signaling | 97166 | 1:1000 |
| ICAM1 | Proteintech | 10831 | 1:1000 |
| Isolectin B4 | Thermo Fisher Scientific | I32450 | 1:100 |
| MCP-1 | Cell Signaling | 81559 | 1:1000 |
| PLCγ1 | Cell Signaling | 2822 | 1:1000 |
| p-Akt | Cell Signaling | 4060 | 1:1000 |
| p-AMPK | Cell Signaling | 2535 | 1:1000 |
| p-eNOS | Cell Signaling | 9571 | 1:1000 |
| p-ERK1/2 | Cell Signaling | 4370 | 1:1000 |
| p-PLCγ1 | Cell Signaling | 2821 | 1:1000 |
| TGFß | Cell Signaling | 3711 | 1:1000 |
| VE-Cadherin | Cell Signaling | 2500 | 1:1000 |
| α-SMA | Abcam | 7817 | 1:500 |

Supplemental Table 1: Antibody Catalog Numbers

Antibodies used in this study are listed along with corresponding manufacturer and catalog numbers. AMPK, 5’ adenosine monophosphate-activated protein kinase; IgG, immunoglobulin G; HRP, horseradish peroxidase; eNOS, endothelial nitric oxide synthase; ERK, extracellular regulated kinase 1/2; FGF1, fibroblast growth factor 1; FGFR1, fibroblast growth factor receptor 1; GAPDH, glyceraldehyde-3-phosphate dehydrogenase; ICAM1, intercellular adhesion molecule 1; MCP-1, monocyte chemoattractant protein-1; PLCγ1, phospholipase Cγ1; TGFß, transforming growth factor beta; VE-cadherin, vascular endothelial cadherin; α-SMA, alpha smooth muscle actin; p-, phosphorylated.
